# The Economic Development of the Nursing Services

**Published:** 1920-12-11

**Authors:** 


### The Economic Development of the Nursing Services.

In the nursing world to-day there is no problem more difficult of solution than the economic development of the trained nurse. It is a question bristling with difficulties and hedged about with prejudice and misunderstanding; it has become a subject for heated discussion and bitter controversy, and yet upon the wide and generous solution of this problem hinges not only the future expansion, but the very existence of the nursing services of to-day. Owing to the peculiar circumstances of their inception the economic status of the nursing services has ever been subservient to their ethical standards, and until recent years the established curricula of the training-schools tended to mental introspection and economic ignorance. Probationers were mainly recruited from that section of the community which provided for the future of its daughters, and the financial interests of the minority were not regarded as worthy of consideration.

*The will to serve* has been, and always will be, the mainspring of the ethical code of the nursing services. It is the tradition from which their work has sprung; it is the origin of that spirit of endeavour from which the nursing services of to-day have been evolved and without which they would crumble to pieces; but *the will to serve* does not necessarily debar the women who serve from receiving for their service a just and generous recompense. The nursing services are essential to the national health, and it is for the nation to realise the necessity to recompense the workers who are serving the national demand. In every branch of service the price of efficiency is high remuneration, the cost of responsibility is adequate compensation; these are the economic laws which govern the work of all who serve humanity, be their calling high or low.

Women drifted into nursing in the days when woman's work was considered of little value in the world, but the policy of drift is dangerous. The nursing services have reached a crucial stage in their development; the will to serve has carried them forward, but it is not in itself sufficient to solve the problems of to-day; individually and collectively the members of the nursing services must evolve the will to understand and the will to apply their understanding. They must convince not only themselves, but the general public, that the maintenance of a high ethical standard is commensurate with a high rate of remuneration, and they must develop their economic capacity so that it is no longer subservient to their ethical code; they must realise that the laws which govern all workers are applicable also to them, for so, and so only, can they hope to attract to the services women who in the future will maintain the traditions which have been created for them by women in the past.

